# Heterotypic Droplet Formation by Pro-Inflammatory S100A9 and Neurodegenerative Disease-Related α‑Synuclein

**DOI:** 10.1021/acs.biomac.5c00130

**Published:** 2025-05-15

**Authors:** Dominykas Veiveris, Aurimas Kopustas, Darius Sulskis, Kamile Mikalauskaite, Mohammad Nour Alsamsam, Marijonas Tutkus, Vytautas Smirnovas, Mantas Ziaunys

**Affiliations:** † Institute of Biotechnology, Life Sciences Center, 54694 Vilnius University, Vilnius LT-10257, Lithuania; ‡ Department of Molecular Compound Physics, Center for Physical Sciences and Technology, Vilnius LT-10257, Lithuania

## Abstract

Liquid–liquid phase separation of proteins and nucleic acids is a rapidly emerging field of study, aimed at understanding the process of biomolecular condensate formation. Recently, it has been discovered that different neurodegenerative disease-related proteins, such as α-synuclein and amyloid-β are capable of forming heterotypic droplets. Other reports have also shown non-LLPS cross-interactions between various amyloidogenic proteins and the resulting influence on their amyloid fibril formation. This includes the new discovery of pro-inflammatory S100A9 affecting the aggregation of both amyloid-β, as well as α-synuclein. In this study, we explore the formation of heterotypic droplets by S100A9 and α-synuclein. We show that their mixture is capable of assembling into both homotypic and heterotypic condensates and that this cross-interaction alters the aggregation mechanism of α-synuclein. These results provide insight into the influence of S100A9 on the process of neurodegenerative disease-related protein LLPS and aggregation.

## Graphical abstract

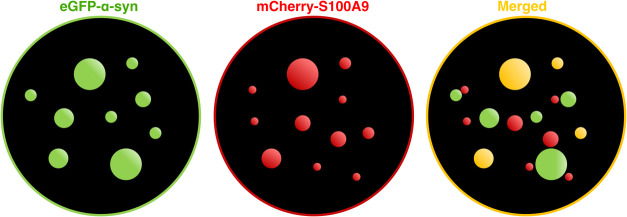

## Introduction

Protein and nucleic acid liquid–liquid phase separation (LLPS) is a process during which biomolecules condense into high concentration membraneless droplets.^1,2^ This phenomenon has recently gained recognition due to its role in various biological processes, including transcription regulation, genome organization and immune response.^1,3–5^ However, recent studies have also shown that aberrant LLPS might be associated with the onset of neurodegenerative disorders, such as Alzheimer’s^6^ or Parkinson’s disease,^7^ as well as various forms of cancer.^3,4,8,9^ Despite enormous progress in this field, the mechanism and implications of LLPS are still far from being fully understood, with new insight being discovered on a regular basis.^10–12^ Due to its function in not only the regulation of countless biological processes, but also manifestation of several widespread diseases, it is imperative to gain a deeper insight into biomolecule condensate formation.

Over the past few years, it has been discovered that a number of different proteins can assemble into heterotypic droplets, i.e., condensates composed of two structurally distinct molecules.^13–15^ In the case of neurodegenerative disorders, this cross-interaction has been hypothesized as a possible intermediate step in the onset of amyloid diseases.^13^ Several amyloidogenic protein pairings were observed to form heterotypic condensates, including α-synuclein (α-syn) with Tau^16,17^ and TDP-43,^18^ as well as prion proteins with Tau^19^ and α-syn.^20^ The amyloid-β peptide^21^ has also been shown to associate into heterotypic droplets with proteins containing low complexity domains.^22^ Most of these protein pairings were reported to cross-interact and affect each other’s aggregation under non-LLPS conditions as well.^15,23–28^ Combined with studies describing amyloid plaques in disease-affected brains having a heterogeneous protein content,^29–31^ it is possible that heterotypic droplet formation plays a critical role in the onset and progression of several neurodegenerative disorders.

In recent years, it has also been discovered that there exists a cross-interaction between α-syn and S100A9.^32^ α-syn is an intrinsically disordered presynaptic protein, whose aggregation into Lewy bodies and Lewy neurites is associated with the second most prevalent neurodegenerative disorder—Parkinson‘s disease.^33–35^ α-syn has been the subject of numerous LLPS studies because of its ability to readily form protein droplets in vitro under high molecular crowding conditions.^7,36,37^ S100A9 is part of a calcium-binding pro-inflammatory S100 protein family.^38^ Due to the protein‘s ability to interact with amyloid-β, α-syn and Tau, as well as its own amyloidogenic properties,^32,39,40^ it is considered that S100A9 may play a critical role in the onset of several neurodegenerative disorders. Recent studies have shown that S100A9 can significantly alter the aggregation pathway of α-syn, leading to the stabilization of a specific fibril secondary structure.^32,41^ In contrast to α-syn, there are currently no reported data on whether S100 family members can undergo LLPS to a comparable extent as other amyloidogenic proteins.

For this reason, our study was dedicated to examining the cross-interaction between α-syn and S100A9 in the context of protein condensate formation. In this work, we demonstrate the ability of α-syn and S100A9 to form both homotypic and heterotypic droplets under high molecular crowding conditions. This cross-interaction influences the aggregation kinetics of α-syn and stabilizes a single fibril conformation. In addition, the resulting strain of fibrils has a notably higher self-replication propensity, when compared to aggregates formed in homotypic α-syn droplets. Combined, these results suggest that heterotypic condensate formation by the pro-inflammatory S100A9 and α-syn is not only possible, but may also be an important factor in the onset of neurodegenerative disorders.

## Materials and Methods

### Cloning

The mCherry and S100A9 genes were amplified and fused using standard PCR methods. The products were inserted into a pET28a(−) vector via NcoI and *Bam*HI restriction sites by standard cloning techniques^42^ yielding mCherry-S100A9 construct with (GGGGS)_2_ linker between the genes and N-terminal (His)_6_ tag. Primers used in this study can be found in Table S1.

### Protein Purification

Recombinant α-syn was purified as described previously.^43^ During the last purification step with size-exclusion chromatography (SEC), the protein was exchanged into PBS (pH 7.4) and stored at −20 °C. After all SEC cycles were completed, the protein fractions were thawed at 4 °C, combined and concentrated to 600 μM using 10 kDa protein concentrators. The prepared protein solutions were then divided into 0.5 mL aliquots and stored at −20 °C prior to use. All experimental procedures in this work were carried out by using the same batch of α-syn. The eGFP-labeled α-syn was purified identically, with the exception of using 70% saturation ammonium sulfate in the protein precipitation step.^44^

S100A9 was purified as described previously.^45^ After gel filtration in PBS buffer (pH 7.4), the protein was concentrated to 500 μM, aliquoted and stored at −80 °C prior to use. mCherry-S100A9 was purified according to the S100A9 protocol with immobilized metal affinity replacing anion exchange chromatography.^41^

### Liquid–Liquid Phase Separation

Poly­(ethylene glycol) (PEG, 20 kDa average molecular weight) was combined with Milli-Q H_2_O and 10× PBS to a final concentration of 40% (w/v) and 1× PBS. pH adjustments to 7.4 were done by adding a concentrated sodium hydroxide solution. Due to the high viscosity of the solution, it was vigorously mixed with magnetic stirring (900 rpm) during the pH measurement procedure. The solution was filtered through a 0.22 μm pore-size syringe filter and stored at 4 °C prior to use.

To induce protein LLPS, the α-syn and S100A9 solutions were combined with 1× PBS (pH 7.4), 40% PEG (pH 7.4) and fluorescently labeled protein stock solutions. The final reaction mixtures contained 20% PEG, 1% labeled protein (either 2 μM eGFP-α-syn or 2 μM mCherry-S100A9), 200 μM α-syn and 0, 50 μM S100A9 concentrations. Control solutions were prepared without the addition of either α-syn or S100A9. Due to the importance of the component mixing order,^46^ the PBS and PEG solutions were combined first, after which the proteins were added (α-syn first, S100A9 s, labeled proteins third). After the addition of each component, the solutions were thoroughly mixed by pipetting for 20 s. Changes in turbidity due to LLPS were visible within the first few seconds. The particle liquid nature was confirmed by tracking rapid droplet fusion events using brightfield microscopy (Supporting Figure S1).

### Droplet Disassembly

α-syn and mCherry-S100A9 stock solutions were combined with 1× PBS (pH 7.4), 40% PEG (pH 7.4) to a mixture containing 240 μM α-syn, 2.4 μM mCherry-S100A9 and 24% PEG. The solution was then incubated for 10 min at 22 °C before being supplemented with 1× PBS and PBS containing either 5 M NaCl (pH 7.4) or 50% (w/v) 1,6-hexanediol (pH 7.4). The resulting final solutions contained 200 μM α-syn, 2 μM mCherry-S100A9, 20% PEG and 750 mM NaCl or 5% 1,6-hexanediol. After an additional 10 min of incubation at 22 °C, the samples were examined using fluorescence microscopy.

### Fluorescence and Brightfield Microscopy

For all microscopy measurements, the samples were first incubated at room temperature (22 °C) for 10 min. Their imaging was then conducted over a span of 15 min at the same temperature. Fifteen μL aliquots of each sample were pipetted onto 1 mm-thick glass slides (Fisher Scientific, cat. No. 11572203), covered with 0.18 mm coverslips (Fisher Scientific, cat. No. 17244914) and imaged as described previously^44^ using an Olympus IX83 microscope with a 40× objective (Olympus LUCPLANFL N 40× Long Working Distance Objective) and fluorescence filter cubes (470–495 nm excitation and 510–550 nm emission filters for eGFP-α-syn or ThT, 540–550 nm excitation and 575–625 nm emission filters for mCherry-S100A9). For fluorescence microscopy images, identical background subtraction and contrast/brightness settings were applied to all images. For brightfield microscopy images, only the contrast/brightness settings were adjusted. Data analysis was done using ImageJ software.^47^

To examine samples containing both fluorescently labeled proteins using two-color fluorescence microscopy, the solutions were placed on cleaned glass coverslips (Menzel Coverslip 24 × 60 mm^2^ #1.5 (0.16–0.19 mm), Thermo Scientific, cat. no. 17244914). For this, a 100 μL droplet suspension containing 200 μM α-syn and 1% of eGFP-α-syn, mCherry-S100A9 or both was slowly added on a bare glass surface using wide-orifice tips (Finntip 250 Wide, Thermo Scientific, cat. no. 9405020) with no pipetting and any subsequent washing of the sample. The miEye, a home-built super-resolution imaging system,^48^ was employed to visualize these fluorescent samples. All experiments were conducted in a TIRF imaging mode with a quad line beamsplitter R405/488/561/635 (F73–866S, AHF Analysentechnik) mounted in the microscope’s body. 488 and 561 nm lasers (Integrated Optics) were used to excite the fluorescently tagged α-syn droplets attached to the glass surface. The emission pathway of miEye was modified to a dual-view regime by inserting a 550 nm long-pass dichroic mirror into the Fourier space present in the microscope’s 4f configuration part. This resulted in the two spectrally distinct emission light collecting channels which, for simplicity, here we refer them to as eGFP channel and mCherry channel. The eGFP channel was equipped with a 525/45 band-pass filter, whereas the mCherry one—with a 697/75 band-pass filter. Both channels were projected and imaged on a single industrial CMOS camera (Alvium 1800 C-511m, Allied Vision Technologies) with its exposure time set to 100 ms. Data analysis was done using ImageJ software,^47^ example is shown as Figure S2.

### Droplet Statistical Analysis

For each condition, a total of thirty 500 × 500 pixel size images (1 pixel—325 nm) were obtained (available at: https://data.mendeley.com/datasets/tvf9nwtdhn/1). The statistical analysis was done as described previously.^46^ In brief, droplet parameters were analyzed with ImageJ software^47^ using automatic threshold selection and particle analysis. All particles of 4 or less pixel size were regarded as artifacts and not taken into account. The total droplet count was the sum of all particles detected in all 30 images for each condition. Average droplet volume was calculated based on the particle areas (assuming completely spherical condensates). Data analysis was done using Origin software and an ANOVA One-way Bonferroni means comparison (*n* = 30).

### Calculation of Heterotypic and Homotypic Droplet Distribution Statistics

For precise alignment of eGFP and mCherry channel images, a calibration sample comprised of carboxylate-modified yellow-green fluorescent polystyrene microspheres (Thermo Fisher, F8811), which were immobilized on poly-l-lysine (Cultrex, cat. no. 3438–100–01)-coated cover glass surface, was imaged in TIRF mode using 488 nm laser for illumination and the same set of band-pass filters (as in fluorescently labeled droplets’ imaging experiments) for cleaning the emitted light and blocking the excitation light. Detection camera’s exposure time was set to 30 ms and its projected pixel size was estimated to be 114.17 nm in XY. Alignment of the two spectrally distinct emission channel images was performed in Fiji 2.16.0 (v1.54p) software^49^ using a dedicated Descriptor-based registration plugin.^50^

After obtaining the exact transformation parameters for aforementioned acquired reference images, the same parameters were used to align the respective images of eGFP-α-syn and mCherry-S100A9 droplets. The aligned images were used for further analysis in Igor Pro 9.0.5.1 (build 56551, WaveMetrics, Inc.) software. Here, individual fluorescent droplets were first marked manually by drawing representative shape figures around their visible contour (regions of interest (ROIs)) with an in-built oval tool. Droplets that appeared deformed and noncircularly shaped or that were overlapping with each other were not included into such analysis. Larger droplets that had visible smaller droplets formed inside them were counted as a singular big entity. The selected ROIs of droplets were then used to generate a binary mask image. All particles present in such masked image were fitted with an ellipse, thus obtaining center position values in X and Y for each separate particle. Lastly, the extracted center coordinates of droplet locations were provided to our custom-written procedure, which calculates the Euclidean distance from every detected particle in eGFP channel to all the ones identified in mCherry channel and then compares the resulting individual distance values with an arbitrarily chosen criterion of colocalization. Here we set this criterion to 500 nm, meaning that the two spectrally distinct droplets which appear to be closer to each other than such distance threshold were accepted as colocalizing particles and considered as a single heterotypic droplet consisting of both eGFP-α-syn and mCherry-S100A9 proteins. The data used for the analysis workflow described here was collected from two independent experiments by acquiring the images of immobilized fluorescent droplets over three different glass surface positions during each experiment. The percent values reported in the main text are the averages of these in total six registered separate fields of view.

### S100A9 Fibril Preparation

S100A9 fibrils were prepared using a previously described protocol,^51^ which generates worm-like amyloid aggregates.^52^ The protein stock solution was diluted to 200 μM using 1× PBS (pH 7.4). S100A9 concentration was determined using a Shimadzu UV-1800 spectrophotometer (ε_280_ = 7100 M^–1^ cm^–1^). The reaction solution was then placed in a 2.0 mL nonbinding test tube (1 mL solution) and incubated under quiescent conditions at 37 °C for 24 h. Fibril formation was determined by atomic force microscopy as described previously^51^ (Figure S5). Aggregate ThT-binding properties were determined by supplementing the fibril solution with 100 μM ThT (from a 10 mM stock solution) and scanning the sample fluorescence emission spectra with a ClarioStar Plus platereader (440 nm excitation, Figure S5). The prepared aggregate solution was then stored at 4 °C. Before further experimental procedures, the fibril solution was concentrated to 400 μM (concentration as monomeric units) by reducing the solution volume in half using 0.5 mL volume 10 kDa Pierce protein concentrators.

An aliquot of the final fibril solution was centrifuged at 12,000*g* for 20 min, after which the protein concentration within the supernatant was determined as described previously. It was observed that the supernatant contained approximately 80 μM S100A9, indicating an equilibrium between large, insoluble aggregates (80% of all protein content) and small oligomers or nonaggregated S100A9 (remaining 20% of all protein content). Additionally, due to fibril adhesion to concentrator membranes and pipet tips, the experimentally used concentration can deviate from the theoretical concentration by up to 5%

### LLPS and Aggregation Kinetics

Solutions containing 20% PEG (w/v), 100 μM thioflavin-T (ThT), 200 μM α-syn and 0, 5, or 50 μM of nonaggregated or fibrillar S100A9 were distributed to 96-well nonbinding plates (100 μL volume in each well, 4 repeats for every condition), sealed with Nunc sealing-tape and incubated under quiescent conditions at 37 °C in a ClarioStar Plus plate reader. Fluorescence intensity measurements were performed every 10 min. ThT fluorescence intensity was monitored using 440 nm excitation and 480 nm emission wavelengths. Due to the time required for sample preparation and distribution procedures, the first measurement was performed approximately 30 min after the mixtures were prepared. During this time, the samples were kept at room temperature (22 °C). All data analysis was done using Origin software.

### Aggregate Reseeding

After the initial LLPS and aggregation reactions, the solutions from each of the 4 repeats were combined and centrifuged at 12,000*g* for 20 min. The supernatants were then carefully removed and replaced with an identical volume of PBS (7.4). The centrifugation and resuspension procedure was repeated three times in order to separate the aggregates from the initial reaction solutions. For the reseeding reactions, the α-syn stock solution was combined with PBS (pH 7.4), ThT and the resuspended aggregates to final reaction mixtures containing 200 μM α-syn, 100 μM ThT and 10% (v/v) aggregate solutions. The reactions were monitored as described previously under quiescent conditions and 37 °C. After 24 h, the samples from each of the 4 repeats were combined and the entire reseeding procedure was repeated for a second time. The final resulting samples were then used for electron microscopy.

### Optical Density Measurements

Samples were placed in a 3 mm path length quartz cuvette and their optical density at 800 nm (OD_800_) was scanned using a Shimadzu UV-1800 spectrophotometer at 22 °C. For each sample, 3 technical repeats were performed and averaged. The values were baseline corrected by subtracting the OD_800_ of 1× PBS.

### Fourier-Transform Infrared Spectroscopy (FTIR)

Fibril samples were centrifuged at 12,000*g* for 20 min. The supernatant was removed and replaced with D_2_O, containing 400 mM NaCl. The centrifugation and aggregate resuspension procedure was repeated 3 times. After the final centrifugation step, the aggregates were resuspended into 50 μL of D_2_O, containing 400 mM NaCl. The sample FTIR spectra were acquired and analyzed as described previously.^51^

### Cryo Electron Microscopy (Cryo-EM)

For cryo-EM sample preparation, 3 μL of α synuclein fibrils were applied to the glow-discharged holey carbon Cu grids (Quantifoil) and blotted with filter paper using Vitrobot Mark IV (FEI Company). The grids were immediately plunge-frozen in liquid ethane and clipped. Cryo-EM data was collected on Glacios transmission electron microscope (Fisher Scientific) operated at 200 kV and equipped with a Falcon IIIEC camera. The micrographs were aligned, motion corrected using MotionCorr2 1.2.1 ^53^ and the contrast transfer function was estimated by CTFFIND4.^54^ The fibrils were picked and all subsequent 2D classifications were performed in Relion 5.0.^55^ Distribution of polymorphs was identified by FilamentTools (https://github.com/dbli2000/FilamentTools) as a part of Relion software. Cryo-EM data collection and 2D classification statistics can be found in Table S2.

## Results

Previous reports of S100A9 and α-syn cross-interactions,^32,41^ as well as the ability of α-syn to form heterotypic condensates,^16–18,20^ has prompted the need to examine this specific protein pairing in the context of liquid–liquid phase separation. In order to test the hypothesis of heterotypic droplet formation, high concentration α-syn samples were combined with a 100-fold lower concentration of either eGFP-α-syn (control) or mCherry-S100A9. To enhance the level of condensate formation, the protein solutions were supplemented with 20% (w/v) of a commonly used molecular crowding agent—poly­(ethylene glycol) (PEG, 20 kDa).^44^ The samples were then imaged using fluorescence microscopy and a total of 30 images for each were obtained and used for particle count and volume distribution analysis. If the hypothesis is correct, both eGFP-α-syn, as well as mCherry-S100A9 should be incorporated into the α-syn droplets and the fluorescence images would show a similar distribution of protein condensates. Oppositely, if mCherry-S100A9 could not interact with α-syn, we would either not be able to detect α-syn droplet formation via mCherry fluorescence or only observe condensates assembled from the labeled protein.

When the samples were analyzed, both α-syn with GFP-α-syn (Figure 1A) and α-syn with mCherry-S100A9 (Figure 1B) solutions contained a large number of droplets with varying size. Surprisingly, analysis of the images revealed that the sample containing mCherry-S100A9 had a significantly higher number of particles (Figure 1F, *n* = 30, *p* < 0.001). However, the average particle volume was not significantly different (Figure 1G, *n* = 30, *p* < 0.001), despite having a lower mean (∼15 μm^3^ as opposed to ∼22 μm^3^). It is worth noting that the apparent particle volume may be influenced by the image acquisition technique and subsequent image processing. To determine if this peculiar effect on the condensate number is not related to the self-assembly of the fluorescently labeled proteins, an identical analysis was conducted on samples containing only 2 μM of eGFP-α-syn or mCherry-S100A9 (Figure 1C,D).

**Figure 1:**
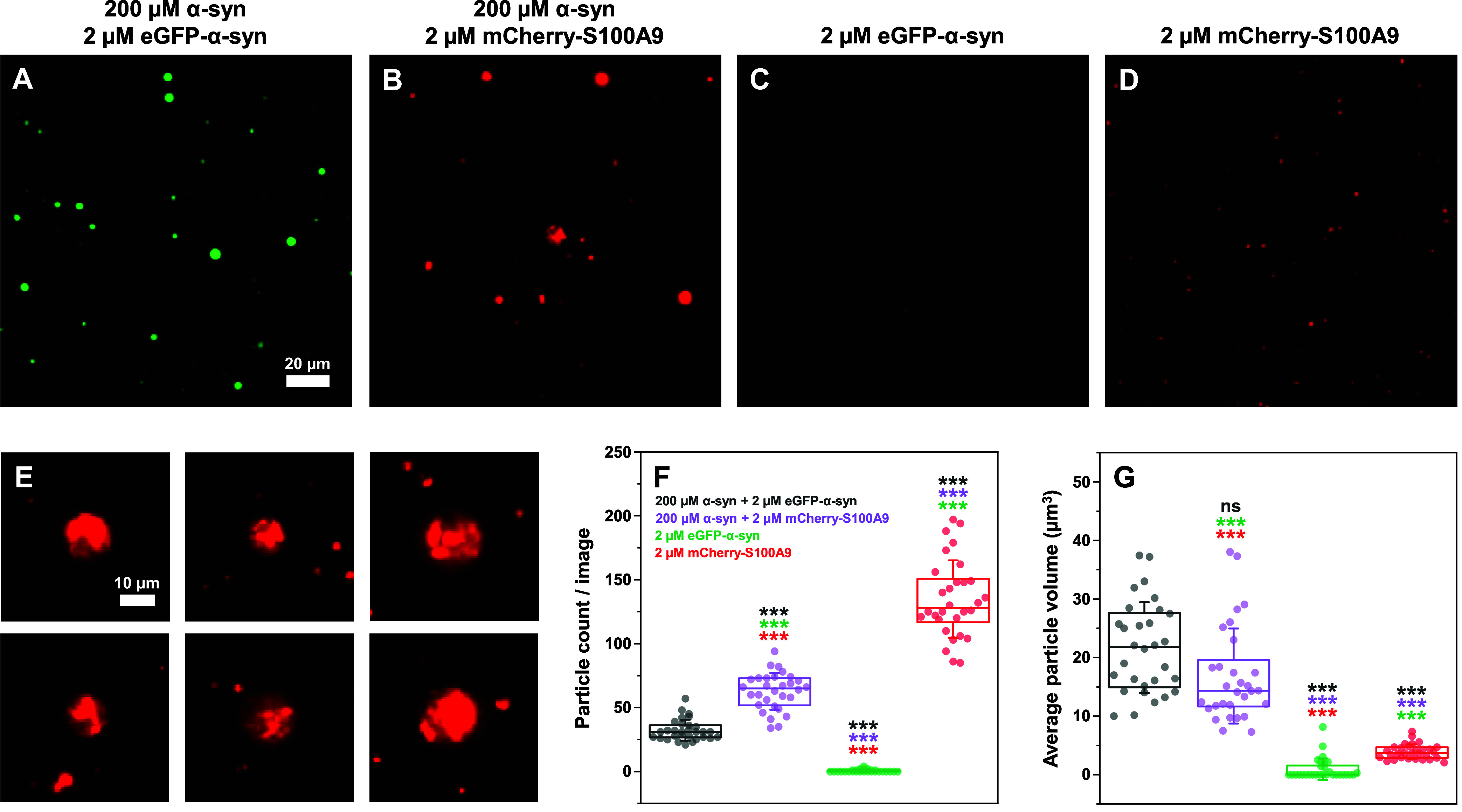
Fluorescence microscopy images of α-synuclein (α-syn) and labeled protein condensate formation. Representative images of 200 μM α-syn with either 2 μM eGFP-α-syn (A) or 2 μM mCherry-S100A9 (B, scale bar—20 μm). Representative control sample images of 2 μM eGFP-α-syn (C) and 2 μM mCherry-S100A9 (D, scale bar—20 μm). Images of unevenly filled droplets from 200 μM α-syn with 2 μM mCherry-S100A9 samples (E, scale bar—10 μm). Statistical analysis (ANOVA Bonferroni means comparison, thirty 500 × 500 pixel size images, ns—not significant, ***-*p* < 0.001) of the particle count (F) and volume (G) per image. Box plots indicate the interquartile range, error bars are for one standard deviation (*n* = 30). All images were acquired after 10 min of sample incubation at 22 °C. Imaging was conducted over a span of 15 min at the same temperature. Additional fluorescence microscopy images (Olympus IX83 microscope) are available as online material.

As expected, eGFP-α-syn formed only a very small number of faintly visible assemblies (due to its low concentration), however, the mCherry-S100A9 sample images contained a large quantity of small particles. Data analysis revealed that the mCherry-S100A9 sample was comprised of a significantly larger number of particles than both α-syn with eGFP-α-syn, as well as α-syn with mCherry-S100A9 samples (*n* = 30, *p* < 0.001). The average particle volume was also significantly lower than in both other samples (Figure 1G). These findings indicate that the fluorescently labeled S100A9 forms visible/detectable particles even at a low concentration. Previous reports have also shown a similar phenomenon for labeled proteins, where the fluorescent tag modulated their LLPS and aggregation propensities.^46^ The self-association of mCherry-S100A9 into small particles, along with their incorporation into α-syn droplets could account for the higher number of condensates detected in the α-syn + mCherry-S100A9 sample. To confirm that mCherry-S100A9 could enter the preformed α-syn droplets, the labeled protein was added after incubating the α-syn sample for 20 min at room temperature (Figure S3).

Another interesting phenomenon observed in the α-syn with mCherry-S100A9 sample was the formation of unevenly filled droplets (Figure 1E). Upon closer inspection, while the droplets had a faintly visible spherical shape, the fluorescently labeled S100A9 was not evenly distributed within them, forming areas of lower and higher fluorescence intensity (Figures 1E, and S3). In contrast, the larger eGFP-α-syn sample droplets all displayed an even fill of the fluorescently labeled protein (Figure S3). These results suggested that, despite the cross-interaction of both proteins during condensate formation, S100A9 still retained a higher tendency to self-associate even within the droplets.

To investigate the nature of these condensates, the α-syn + mCherry-S100A9 solutions (Figure 2A) were supplemented with either 0.75 M NaCl (Figure 2B) or 5% 1,6-hexanediol (Figure 2C) to disrupt electrostatic and hydrophobic interactions between proteins.^56^ In both cases, there was a significant (*n* = 30, *p* < 0.001) reduction in the number of observable particles (Figure 2E). The effect was most prevalent in the case of the additional 0.75 M NaCl, suggesting a stronger role of electrostatic interactions in droplet formation. Interestingly, the smaller volume droplets were more susceptible to the effect of both additives, which resulted in an increase of the average particle volume per image (Figure 2F). However, this change was only statistically significant in the case of hexanediol (*n* = 30, *p* < 0.01), where it also caused part of the particles to become deformed (Figure 2D).

**Figure 2:**
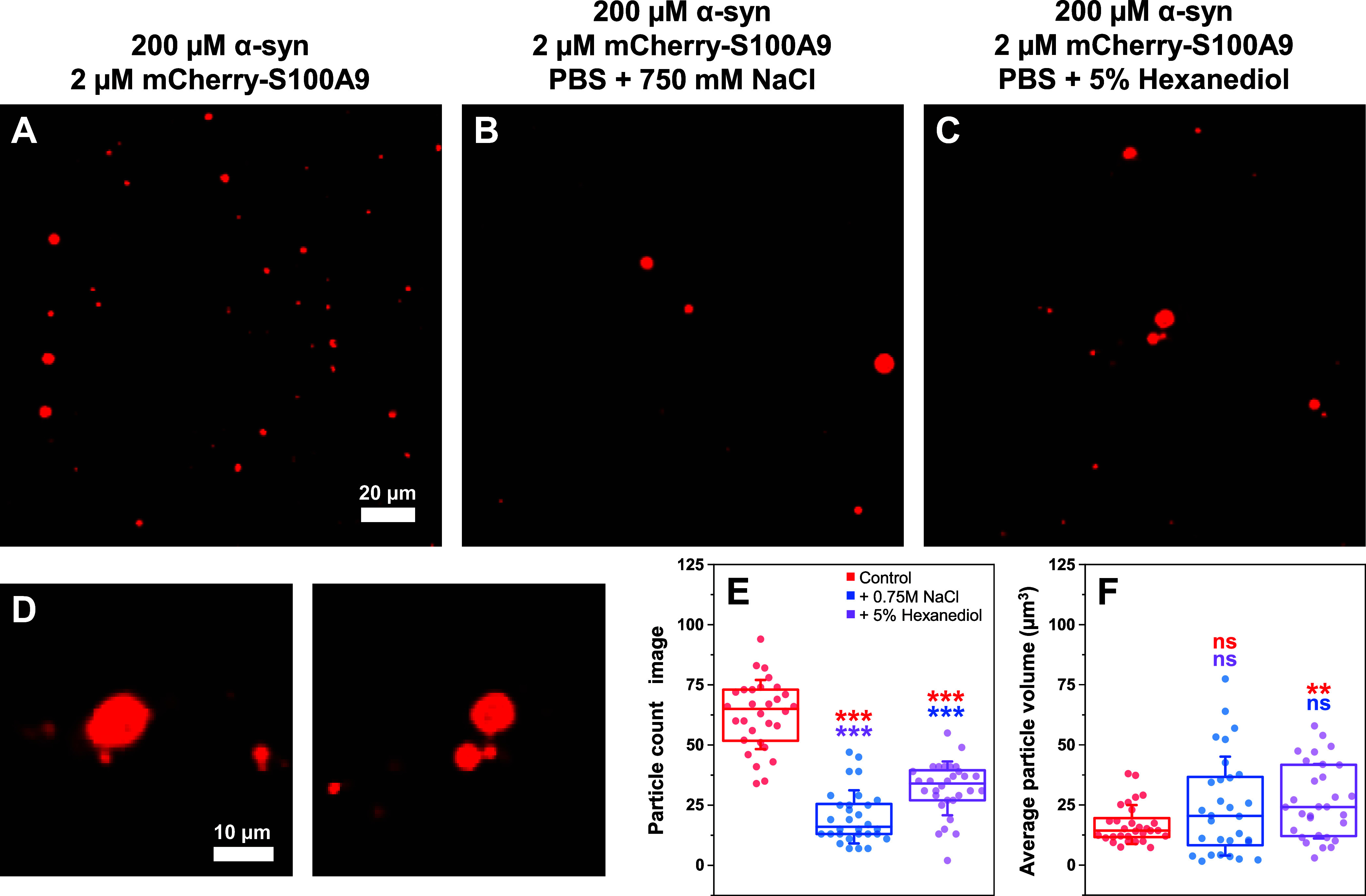
Condensate disassembly by high ionic strength or 1,6-hexanediol. Images of 200 μM α-syn with 2 μM mCherry-S100A9 in the absence (A) and presence of additional 750 mM NaCl (B) or 5% 1,6-hexanediol (C). Images of deformed droplets found under 5% 1,6-hexanediol conditions (D). Statistical analysis (ANOVA Bonferroni means comparison, thirty 500 × 500 pixel size images, ns—not significant, **-*p* < 0.01, ***-*p* < 0.001) of the particle count (E) and volume (F) per image. Box plots indicate the interquartile range, error bars are for one standard deviation (*n* = 30). NaCl and 1,6-hexanediol were added to the solutions after 10 min of incubation at 22 °C. Images were acquired after an additional 10 min of incubation under identical conditions. Imaging was conducted over a span of 15 min at the same temperature.

In order to determine if the protein condensates were all heterotypic, or if the droplets could also be homotypic, samples containing α-syn with both fluorescently labeled proteins were examined using two-color fluorescence microscopy. Overlaid two-color images revealed perfect colocalization, and varying levels of either protein (Figure 3A–C). The majority of small particles were composed mainly of mCherry-S100A9, while generally larger droplets contained either only eGFP-α-syn or both labeled proteins. A statistical analysis of several surface locations revealed that (44.7 ± 3.2) % particles were heterotypic (*n* = 2581). Out of the remaining condensates, (43.1 ± 2.5) % droplets contained only mCherry-S100A9 and the remaining (12.2 ± 2.5)%—only eGFP-α-syn.

**Figure 3:**
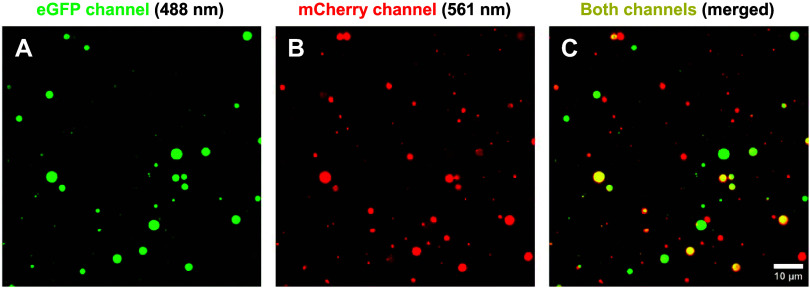
TIRF microscopy images of the surface-immobilized α-syn and α-syn-eGFP or mCherry-S100A9 droplets. Using a 488 nm laser illumination (miEye microscope), the fluorescence of eGFP-α-syn and mCherry-S100A9 was observed in the eGFP channel (A), while exciting this sample with a 561 nm laser yielded the fluorescence visible in the mCherry channel (B). These two images were acquired on the same surface position of the sample. Overlaid eGFP and mCherry channel images (C) show the colocalization of such droplets at the same surface location. All images were acquired after 10 min of sample incubation at 22 °C. Imaging was conducted over a span of 15 min at the same temperature.

Since the proteins were able to form heterotypic droplets, further examination was conducted to determine how different concentrations of unlabeled S100A9 would affect α-syn LLPS. When 2 μM S100A9 was present in solution, there were no statistically significant differences in either the particle count or volume distributions from the control (Figure 4A,B,F,G). The presence of 50 μM S100A9, however, resulted in a significantly higher number of particles (Figure 4C,F). The sample average volume distribution followed a similar trend as in the case of α-syn with mCherry-S100A9 (Figure 1G), where the sample with both proteins had a lower, yet not significantly different mean value (Figure 4G).

**Figure 4:**
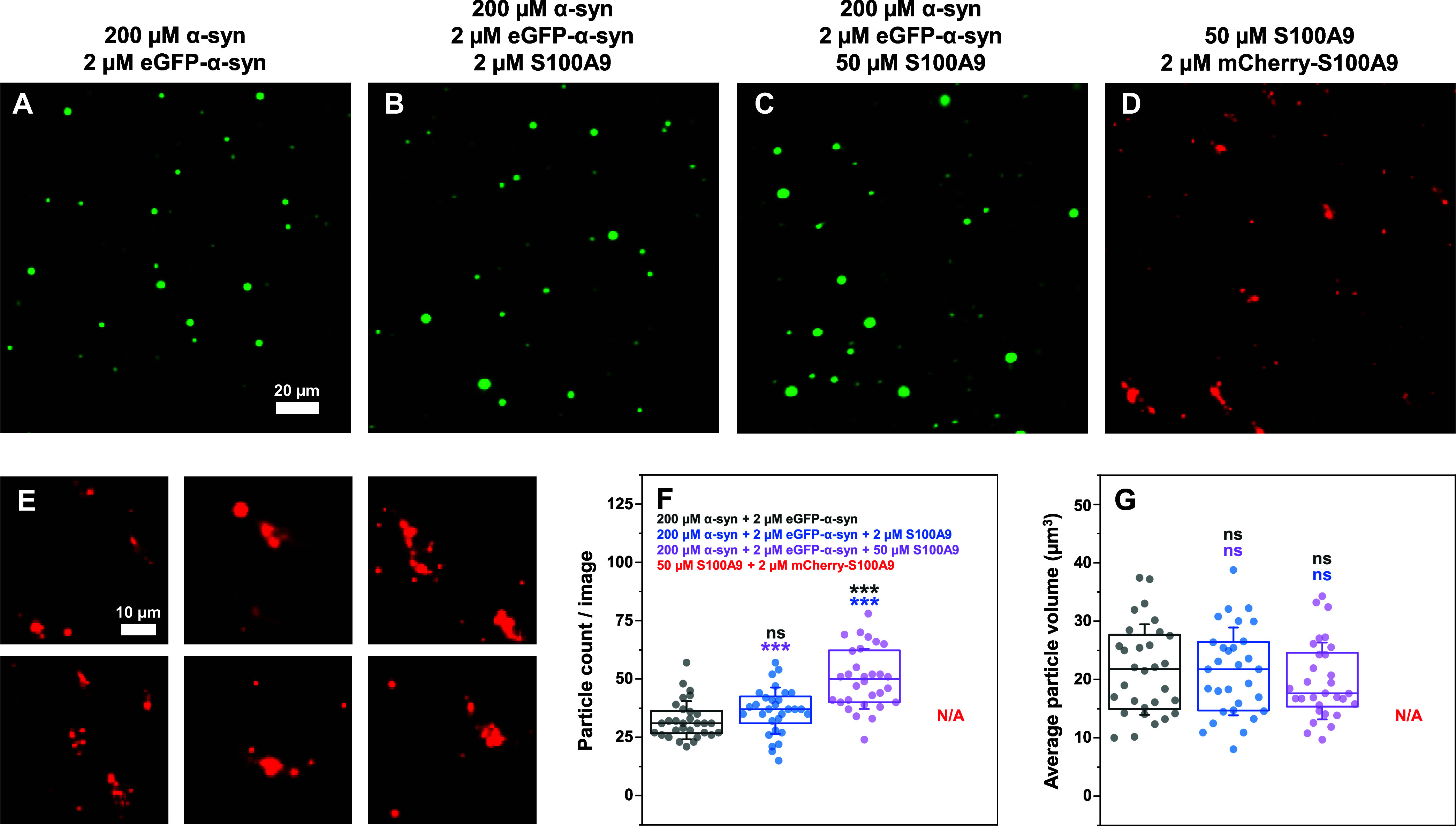
Fluorescence microscopy images of α-syn and S100A9 condensate formation. Images of 200 μM α-syn with 2 μM eGFP-α-syn (A), 200 μM α-syn with 2 μM eGFP-α-syn and 2 μM S100A9 (B), 200 μM α-syn with 2 μM eGFP-α-syn and 50 μM S100A9 (C) or 50 μM S100A9 with 2 μM mCherry-S100A9 (D, scale bar—20 μm). Images of droplets and aggregates from 50 μM S100A9 with 2 μM mCherry-S100A9 samples (E). Statistical analysis (ANOVA Bonferroni means comparison, thirty 500 × 500 pixel size images, ns—not significant, ***-*p* < 0.001) of the particle count (F) and volume (G) per image. Box plots indicate the interquartile range, error bars are for one standard deviation (*n* = 30). The 50 μM S100A9 with 2 μM mCherry-S100A9 images contained both droplets and aggregates, which prevented an accurate statistical analysis. All images were acquired after 10 min of sample incubation at 22 °C. Imaging was conducted over a span of 15 min at the same temperature. Additional fluorescence microscopy images (Olympus IX83 microscope) are available as online material.

Interestingly, when the sample did not contain α-syn, S100A9 with mCherry-S100A9 formed a mixture of droplets and various amorphous aggregates (Figure 4E). The same was true when the samples only contained unlabeled S100A9, where small droplets and amorphous structures were observed with brightfield microscopy (Figure S4). The presence of these structures prevented an accurate statistical analysis and also raised questions regarding the nature of the cross-interaction between both proteins. Taking into consideration that the sample with both proteins contained a significantly larger number of droplets and no visible aggregates, there existed a number of possible explanations. First, the cross-interaction between both proteins could stabilize S100A9 and prevent its aggregation, which would explain the lack of amorphous structures and a higher number of droplets. Second, the S100A9 aggregates may be present in the sample, but they are not visible due to their inability to interact with eGFP-α-syn. Lastly, S100A9 may associate with α-syn into droplets and then rapidly form aggregates, which would explain the previously observed uneven distribution in part of the condensates (Figure 1E).

To answer this question, S100A9 was aggregated into fibrils prior to being combined with α-syn and eGFP-α-syn. During sample analysis with brightfield microscopy, the first notable observation was that S100A9 fibrils, which are normally short worm-like structures (Figure S5),^52^ associated into large aggregate clusters (Figure 5A). Despite this high-level of self-assembly, the S100A9 fibrils retained their ThT-binding propensity (Figure S6). These S100A9 aggregate assemblies ranged from several to well over a hundred micrometers in size. Another interesting factor was that the S100A9 aggregates were clearly visible during fluorescence microscopy due to their association with eGFP-α-syn (Figure 5B). These results indicate that the hypothesis of S100A9 aggregation outside of α-syn droplets in the protein mixture is not correct, as they would be clearly visible in the fluorescence microscopy images. The possibility of rapid S100A9 aggregation within heterotypic droplets remains inconclusive, however, the observed large size of S100A9 structures under high molecular crowding conditions also makes this event unlikely. Additionally, the fluorescence microscopy images revealed that there is a high level of cross-interaction between α-syn and S100A9 aggregates.

**Figure 5:**
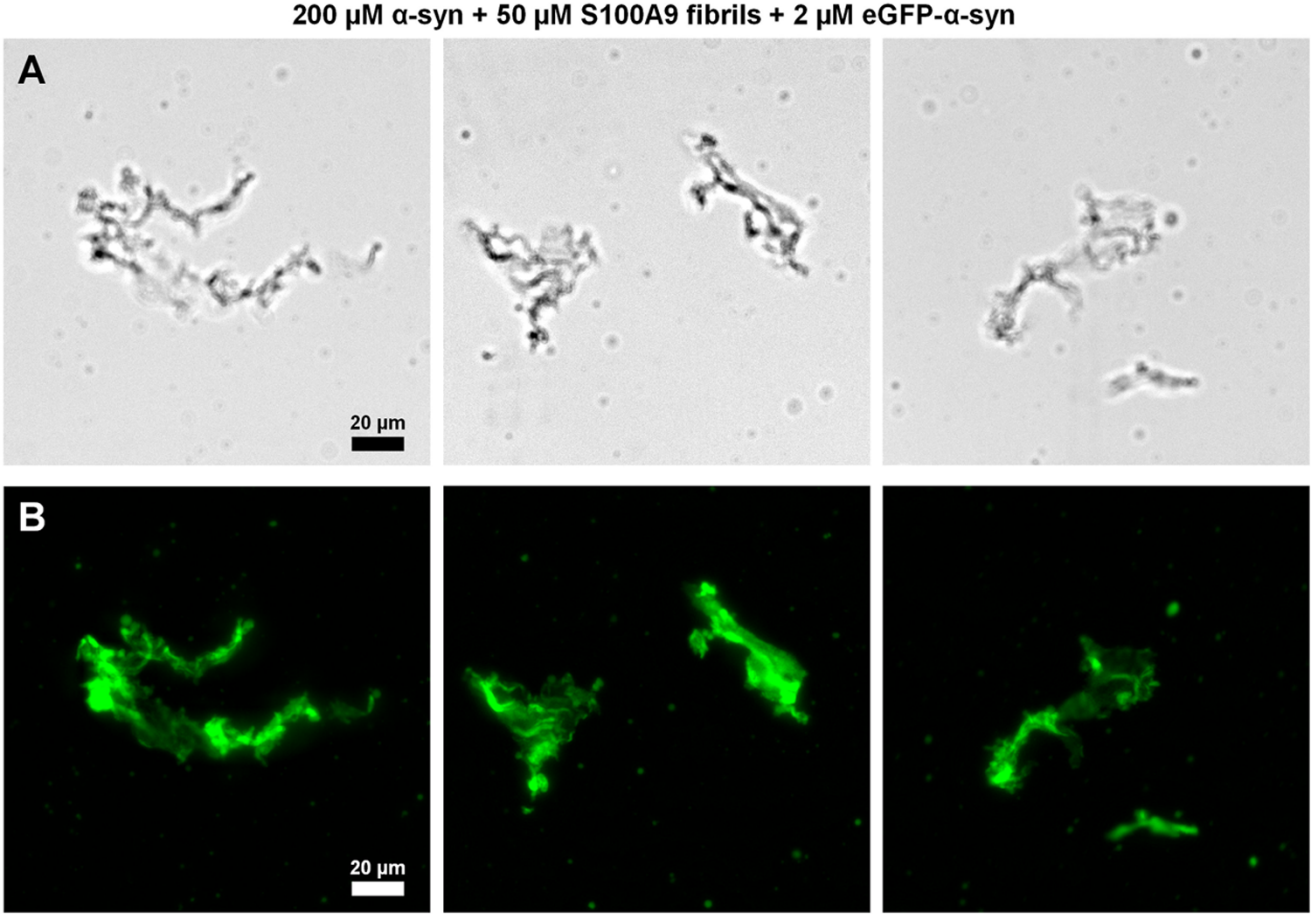
Brightfield and fluorescence microscopy images of S100A9 aggregates with α-syn. Brightfield microscopy images (Olympus IX83 microscope) of samples containing 200 μM α-syn, 50 μM S100A9 fibrils and 2 μM eGFP-α-syn (A, scale bar—20 μm). Fluorescence microscopy images of the samples at the same exact positions (B, scale bar—20 μm) All images were acquired after 10 min of sample incubation at 22 °C. Imaging was conducted over a span of 15 min at the same temperature. The prepared S100A9 fibril stock samples contained an equilibrium between large, insoluble aggregates (80%) and small oligomers or nonaggregated S100A9 (20%).

Since the results of this study indicated that both native, as well as aggregated S100A9 can interact with α-syn under LLPS-inducing conditions, it was further investigated whether this cross-interaction can influence the process of α-syn fibril formation. Samples containing different concentrations of S100A9 and its aggregated form with or without α-syn were monitored under LLPS-inducing conditions with the use of an amyloid-specific dye—thioflavin-T (ThT). In the case of the S100A9 control samples, the native protein either displayed a very low increase in ThT fluorescence intensity (at 5 μM concentration, Figure 6A,C) or had a rapid initial increase, followed by a second change in fluorescence intensity (at 50 μM concentration, Figure 6A,C). The double transition in ThT fluorescence intensity can be attributed to the initial assembly of droplets/aggregates, after which amyloid fibrils are formed.^44^

**Figure 6:**
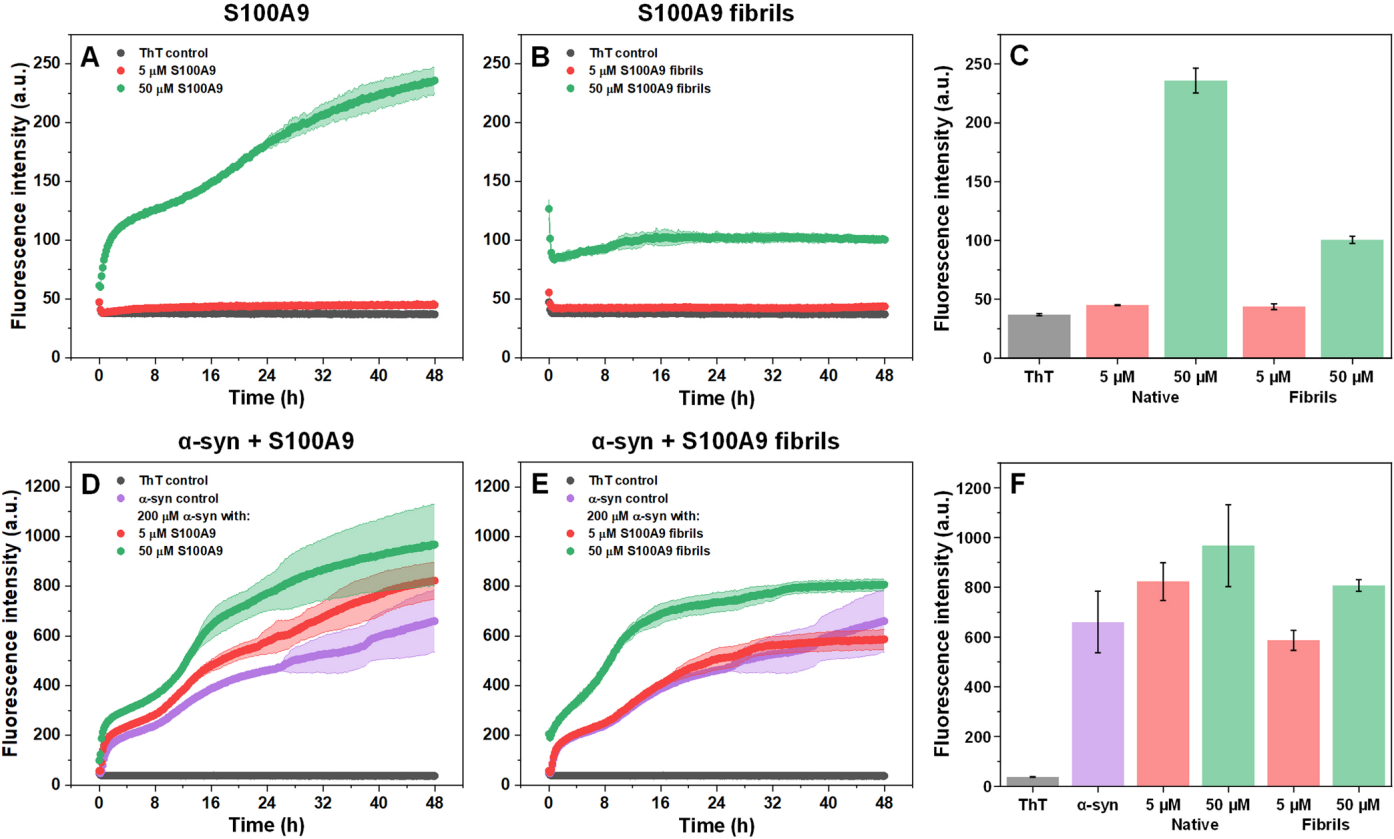
LLPS and aggregation kinetics of α-syn with S100A9. Native S100A9 (A), S100A9 fibril (B), α-syn with native S100A9 (D) and α-syn with S100A9 fibrils (E) sample ThT fluorescence intensity changes over 48 h of incubation under LLPS-inducing conditions. End-point fluorescence intensity values of samples after 48 h of incubation (C, F). Error plots and bars are for one standard deviation (4 technical repeats for each condition). S100A9 fibril stock samples contained an equilibrium between large, insoluble aggregates (80%) and small oligomers or nonaggregated S100A9 (20%).

The S100A9 fibril stock samples (prepared under non-LLPS conditions) contained an equilibrium between large, insoluble aggregates (80%) and small oligomers or nonaggregated S100A9 (20%). When they were subjected to the same conditions as the native S100A9 (Figure 6B), the 50 μM sample end-point fluorescence intensity values were only half of what was observed in the case of the native protein sample (Figure 6C). This result has a few possible explanations. First, the LLPS conditions may alter the aforementioned equilibrium between the different protein states. Second, the LLPS conditions cause the native protein to form S100A9 aggregates with a slightly different secondary structure (Figure S7), which may possess distinct ThT-binding properties.^57^ Lastly, the preformed S100A9 fibrils tend to associate into larger clusters due to the increase in molecular crowding (Figure S6), leading to ThT self-quenching.

When α-syn was combined with 5 or 50 μM of native S100A9, the ThT intensity kinetic curves followed a similar double-sigmoidal trend, with the only exception being the fluorescence intensity values (Figure 6D). In the case of S100A9 fibrils, the kinetic curves of the α-syn control and 5 μM aggregated S100A9 were nearly identical (Figure 6E). Surprisingly, the second increase in ThT fluorescence intensity occurred much quicker when the sample contained 50 μM S100A9 fibrils, which may be related to aggregate surface-enhanced secondary nucleation.^58^ This sample also yielded a higher end-point fluorescence intensity value, similar to the one with native S100A9 (Figure 6F). The differences between these end-point intensity values can stem from different fibril concentrations, S100A9 aggregate assembly or the formation of structurally distinct α-syn fibrils, as was previously shown with this protein pairing.^41^ In order to figure this out, the α-syn samples were subjected to two rounds of reseeding. As self-replication is a hallmark of amyloid structures, the two rounds of reseeding (10% seed) would simultaneously increase the abundance of α-syn fibrils, greatly diminish the number of amorphous aggregates, as well as massively reduce the concentration of S100A9 fibrils and PEG in the samples. The reseeding procedure was done under non-LLPS conditions, in order to avoid the spontaneous formation of LLPS-induced aggregates.

During the first round of reseeding, α-syn fibrils (initially prepared with 5 or 50 μM native S100A9) both displayed exponential aggregation curves (Figure 7A), which were similar in shape to the control and indicated efficient reseeding. Both samples had a much larger end-point fluorescence intensity than the control (Figure 7C), which supported the hypothesis that native S100A9 can lead to the formation of a higher concentration of α-syn fibrils or modulate their resulting structures. Oppositely, α-syn aggregates formed in the presence of S100A9 fibrils produced a lower end-point fluorescence intensity than the control (Figure 7B,C), suggesting that either the protein structures had a different ThT-binding mode or that the self-replication was highly inefficient.

**Figure 7:**
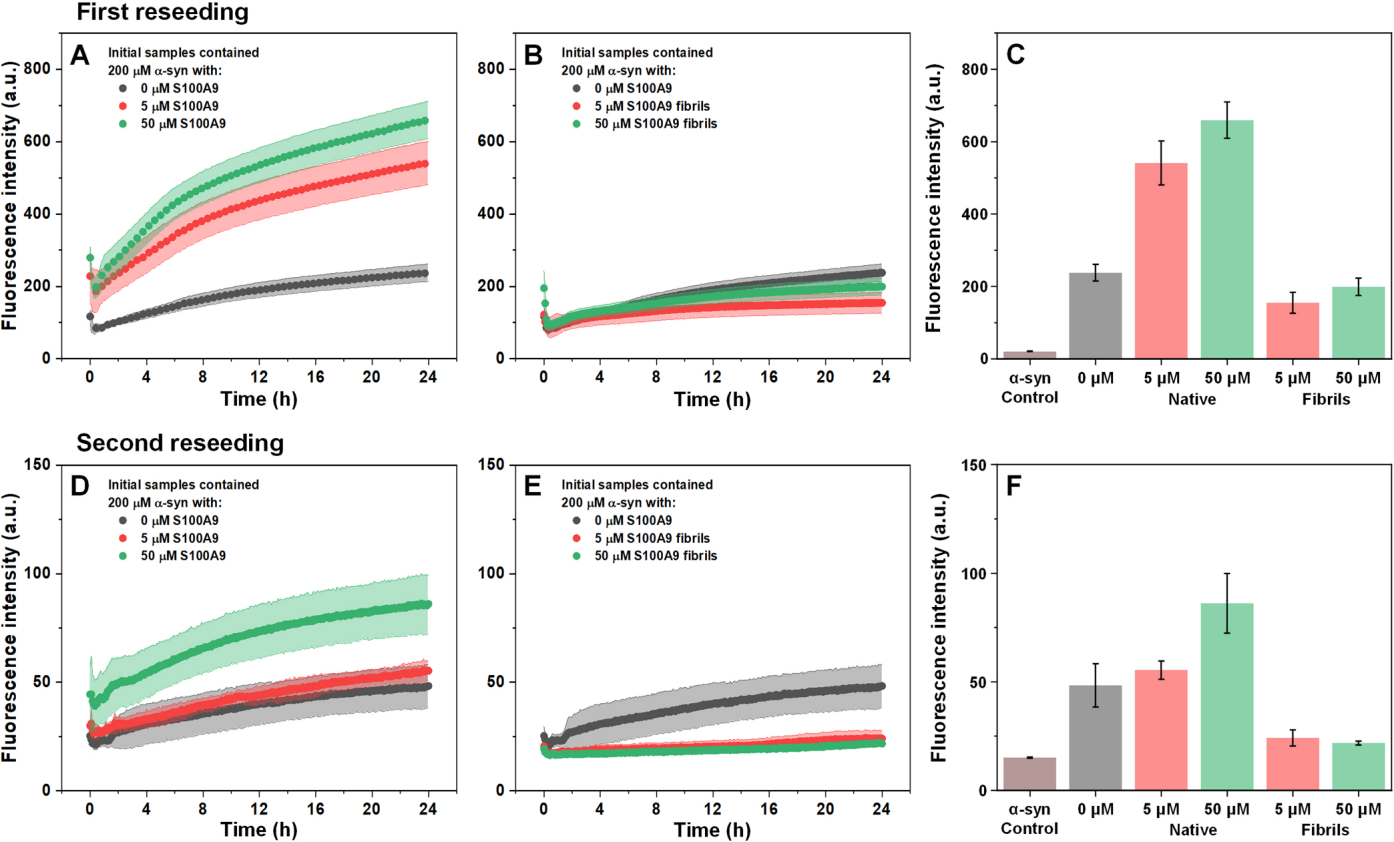
α-Syn aggregate reseeding kinetics and end-point fluorescence intensity values. First and second round of α-syn aggregate reseeding after their preparation in the presence of native (A, D) or aggregated (B, E) S100A9. End-point fluorescence intensity values of the first (C) and second (F) round of reseeding (monomeric α-syn sample intensity added for comparison). Error plots and bars are for one standard deviation (6 technical repeats for each condition). Larger scale panel (E) kinetics are available as Figure S8.

The second round of reseeding resulted in an even higher disparity between the results. In the case of α-syn aggregates prepared in the presence of native S100A9, only the sample which initially contained 50 μM S100A9 resulted in a notable increase in ThT fluorescence intensity, while the 5 μM initial S100A9 sample was similar to the control (Figure 7D,F). As in the first reseeding, the ThT fluorescence intensity increase of samples initially prepared with aggregated S100A9 followed a similar trend (Figure 7E,F). The sample signal intensity was only slightly higher than the α-syn monomer control and much lower than the α-syn aggregate control (Figure S8). These results indicated that α-syn aggregates were only capable of efficient self-replication when they were initially prepared in the absence of S100A9 or in combination with native S100A9.

To evaluate what structural differences occurred due to the formation of heterotypic α-syn and S100A9 droplets, the α-syn control and α-syn with 50 μM S100A9 samples (after two rounds of reseeding) were selected for further investigation by Cryo-EM. Out of the three, the fibril content of the sample initially prepared with S100A9 aggregates was insufficient for an accurate determination of their structures. In the case of the control sample, it contained two structurally distinct fibrils.The dominant type was twisted fibrils with a cross-sectional width of 70 Å and constituted approximately 92% of the total distribution (Figures 8A,C, and S9). The remaining 8% were composed of nontwisted aggregates with a cross-sectional width of 100 Å. When the initial α-syn fibrils were prepared in the presence of 50 μM S100A9, the entire reseeded sample consisted of fibrils with an identical morphology (Figures 8B and S10). This dominant fibril class was the same under both conditions, indicating that the presence of native S100A9 can reduce the variability of α-syn aggregate formation.

**Figure 8:**
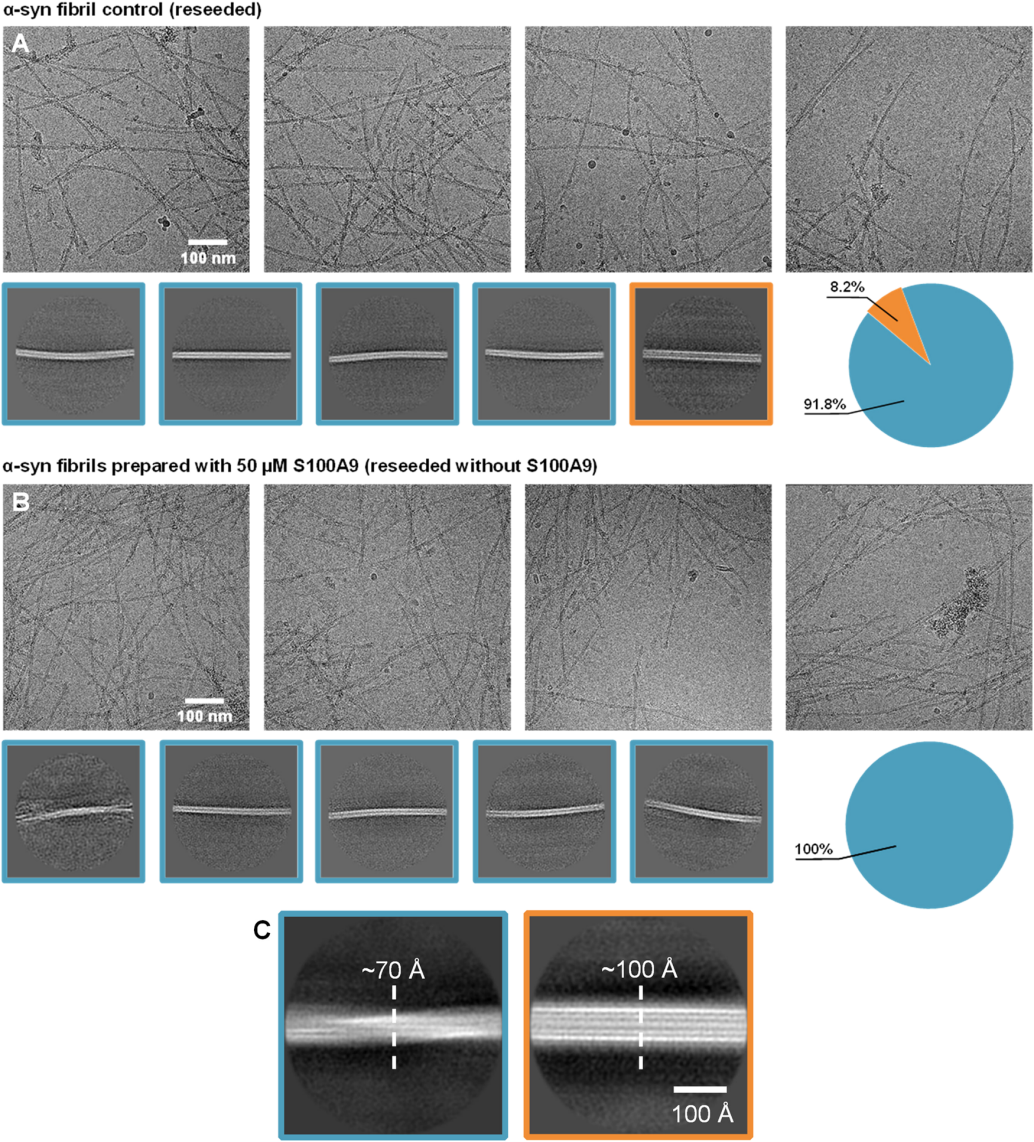
Cryo-EM images of α-syn fibrils after two rounds of reseeding. Images of α-syn fibrils which were prepared in the absence (A) or presence (B) of 50 μM native S100A9 and then reseeded twice (10% seed (v/v) each time). Close-up images of both fibril classes and their cross-sectional widths (C). Different fibril classes are indicated by blue and orange outlines. Fibril distributions are shown as color-coded pie-charts next to the images.

## Discussion

The cross-interaction between S100A9 and α-syn has previously been observed to modulate the amyloid formation process of α-syn in vitro,^41^ as well as their tendency to colocalize in aggregate plaques in vivo.^32^ In this work, we examined whether this interaction could also lead to the formation of heterotypic protein condensates, a phenomenon previously observed for certain sets of amyloidogenic proteins. We discovered that α-syn and S100A9 can associate into condensates under high molecular crowding conditions and observed several interesting aspects regarding their liquid–liquid phase separation.

Previous reports of amyloid protein heterotypic droplet formation have generally shown a homogeneous distribution of both molecules within condensates.^17,19,20^ This did not appear to be the case in respect to α-syn and S100A9, where we observed the formation of regular homotypic droplets alongside their heterotypic variants. Fluorescence microscopy images of α-syn samples containing mCherry-S100A9 also displayed an uneven distribution of the labeled protein within the condensate. These findings would suggest that both proteins can exist within the same droplets, however, they may have a higher level of affinity toward self-association.

Additionally, a number of interesting observations were made in the case of S100A9 within a high molecular crowding environment. Both the fluorescently labeled and regular S100A9 displayed a very high level of self-association under LLPS-inducing conditions, including the formation of droplets and aggregates. We have previously observed that fluorescently labeled proteins can possess a higher level of condensate formation,^46^ however, this effect appeared to be exceptionally high for mCherry-labeled S100A9. The high molecular crowding environment also had an interesting influence on S100A9 aggregates. While they typically exist as short worm-like structures, the LLPS-inducing conditions caused them to clump into large aggregate structures. These assemblies were highly efficient at binding α-syn and even enhancing their transition to amyloid fibrils via surface-mediated nucleation.

Another interesting aspect of this cross-interaction was the stabilization of a single dominant α-syn fibril strain. Such an effect of S100A9 has previously been observed in vitro, where the mixture of both proteins resulted in the generation of a single α-syn aggregate secondary structure.^41^ Taking into consideration that only a part of S100A9 and α-syn formed heterotypic droplets, it is likely that this influence on the resulting fibril structure is not entirely dependent on the protein association within droplets. Since the relative abundance of α-syn aggregation centers is low during the process of unseeded nucleation, the presence of even a low concentration of S100A9 within or outside of the condensates may influence nuclei formation and elongation.

Based on previously reported work, there are a few possible explanations for this cross-interaction and its effect on α-syn aggregation. In the work by Horvath et al. it was shown that the C-terminal part of α-syn can bind with S100A9, which causes the intrinsically disordered protein to rapidly form amyloid structures.^32^ Oppositely, studies by Toleikis et al. reported that the N-terminal region of α-syn interacts with the S100A9 interface comprised of Helix 1, Helix 4 and 86–96 residues.^59^ Additionally, S100A9 has been shown to possess chaperone-like properties by suppressing amyloidogenic protein primary nucleation.^60^ In the case of S100A9 fibrils, they may serve as a hydrophobic surface for α-syn nucleation^58^ and also promote the formation of a specific fibril type. These transient interactions and inhibition or promotion of α-syn conformational transitions into certain types of amyloid nuclei may contribute to our observed heterotypic LLPS, as well as kinetic results.

The reseeding experiments also revealed an unexpected result of α-syn and S100A9 heterotypic droplet formation. When both proteins were in their native state within the condensate, α-syn aggregated into fibrils which pertained a notably higher level of self-replication properties when subjected to a non-LLPS-inducing environment. Taking into consideration the localization of both proteins, as well as the high molecular crowding environment in vivo, it is possible that heterotypic droplet formation results in fibrils with a higher tendency for self-replication. This would suggest that heterotypic droplet formation between the two proteins may be a critical step in the onset and progression of α-syn-related neurodegenerative disorders.

## Conclusions

The pro-inflammatory S100A9 and neurodegenerative disease-related α-synuclein are capable of forming heterotypic droplets under LLPS-inducing conditions. This cross-interaction can lead to a stabilization of a specific α-syn fibril strain, which is capable of effective self-replication under non-LLPS conditions. The ability of these proteins to form heterotypic condensates complements the previously reported cross-interactions in vivo and in vitro, adding another piece to the complex amyloid interactome puzzle.

## Supplementary Material



## Data Availability

All raw data and additional images are available in an online data repository at: https://data.mendeley.com/datasets/tvf9nwtdhn/1.

## Notes

microscopy images of α-synuclein droplet fusion events; S100A9 and α-synuclein heterotypic droplet formation; S100A9 distribution within droplets; S100A9 condensate formation and S100A9 aggregates with either ThT or eGFP-α-synuclein; optical density of S100A9 and α-synuclein samples; atomic force microscopy image of S100A9 aggregates, bound-ThT fluorescence spectra; FTIR spectra of S100A9 fibrils; α-synuclein reseeding kinetics and hierarchical classifications of α-synuclein aggregates; sequences of primers used, as well as Cryo-EM data statistics (PDF)
