# Supplementary material for: Heterotypic Droplet Formation by Pro-Inflammatory S100A9 and Neurodegenerative Disease-Related α‑Synuclein
Source: Biomacromolecules. 2025 May 15;26(6):3525–37. doi: 10.1021/acs.biomac.5c00130 (PMC12152929; doi:10.1021/acs.biomac.5c00130)
Supplement: Supplementary file 1 [file bm5c00130_si_001.pdf]

Heterotypic droplet formation by pro-inflammatory S100A9 and neurodegenerative disease-related alpha-synuclein

Dominykas Veiveris[1#], Aurimas Kopustas[1,2#], Darius Sulskis[1], Kamile Mikalauskaite[1], Mohammad Nour Alsamsam[1,2], Marijonas Tutkus[1,2], Vytautas Smirnovas[1], Mantas Ziaunys[1*]

*Correspondence: mantas.ziaunys@gmc.vu.lt

[#] Authors contributed equally

[1]Institute of Biotechnology, Life Sciences Center, Vilnius University, Vilnius, Lithuania

[2]Department of Molecular Compound Physics, Center for Physical Sciences and Technology,

Vilnius, Lithuania

Supporting information

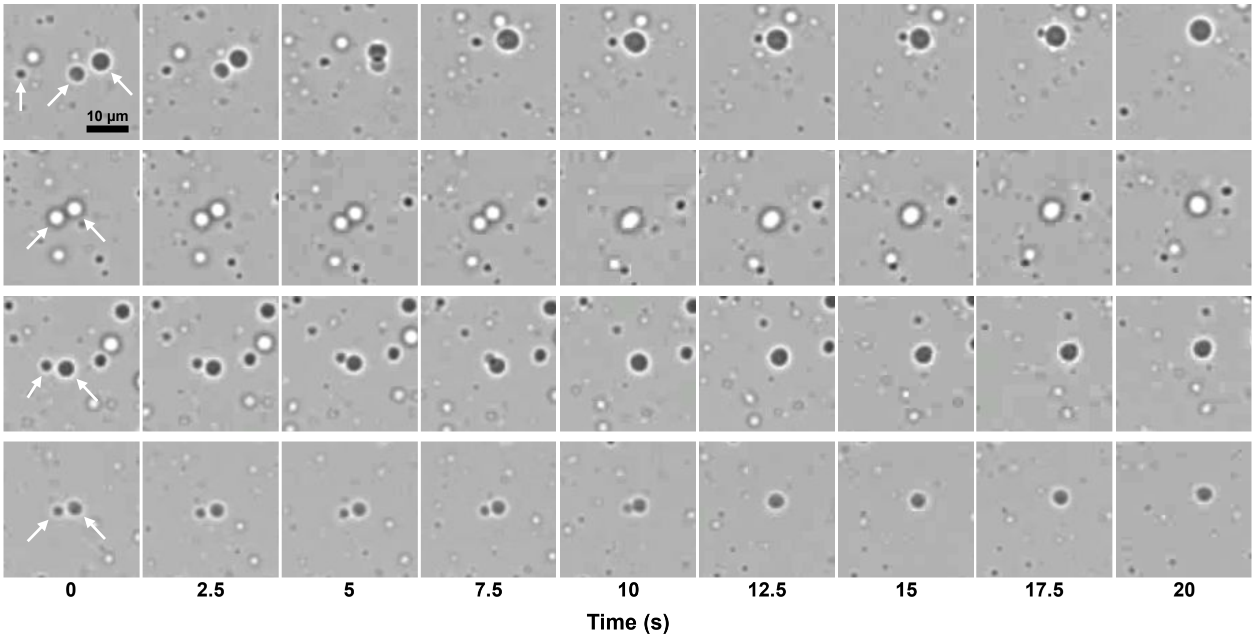

Figure S1. Alpha-synuclein droplet fusion events monitored by brightfield microscopy. Scale bar (10 μm) is identical for all images.

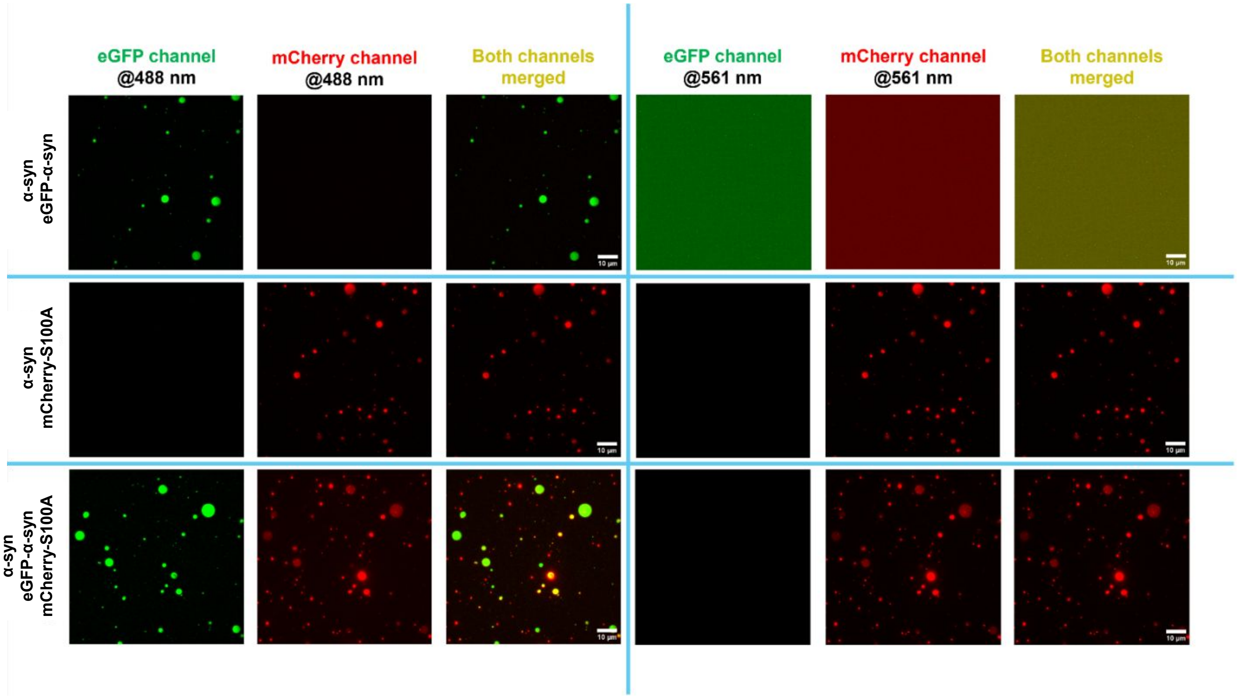

Figure S2. TIRF microscopy images of droplets formed from α-syn with either eGFP-α-syn, mCherry-S100A9 or both. Such droplets were immobilized on the glass surface and visualized in the eGFP and mCherry spectral channels using either 488 nm or 561 nm laser excitation. For each fusion construct, the same imaged surface position is shown and every set of images separated by the blue lines have identical minimum and maximum intensity scale values applied to them.

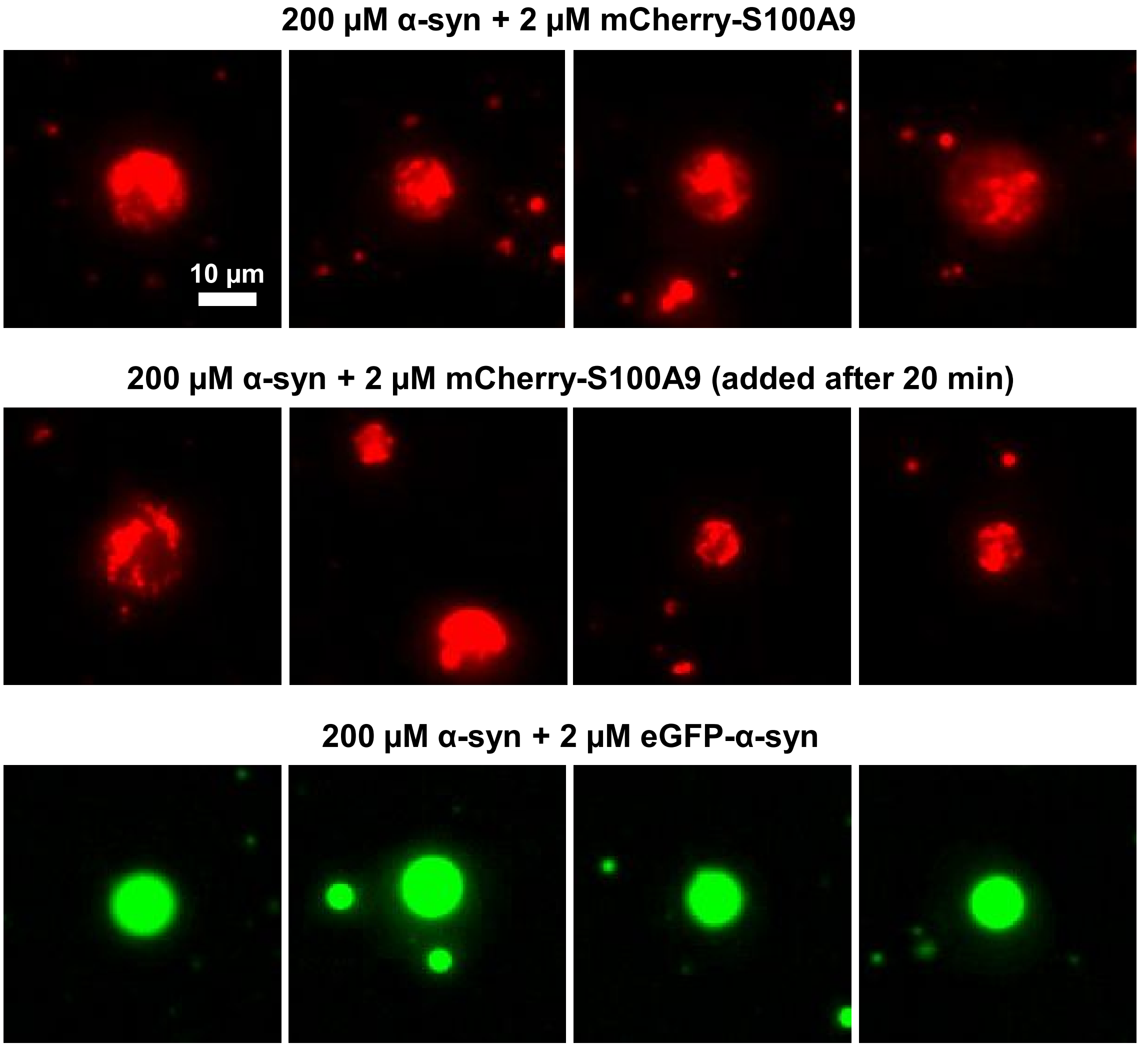

Figure S3. Comparison of labeled protein distribution within α-syn droplets. Scale bar (10 μm) is identical for all images.

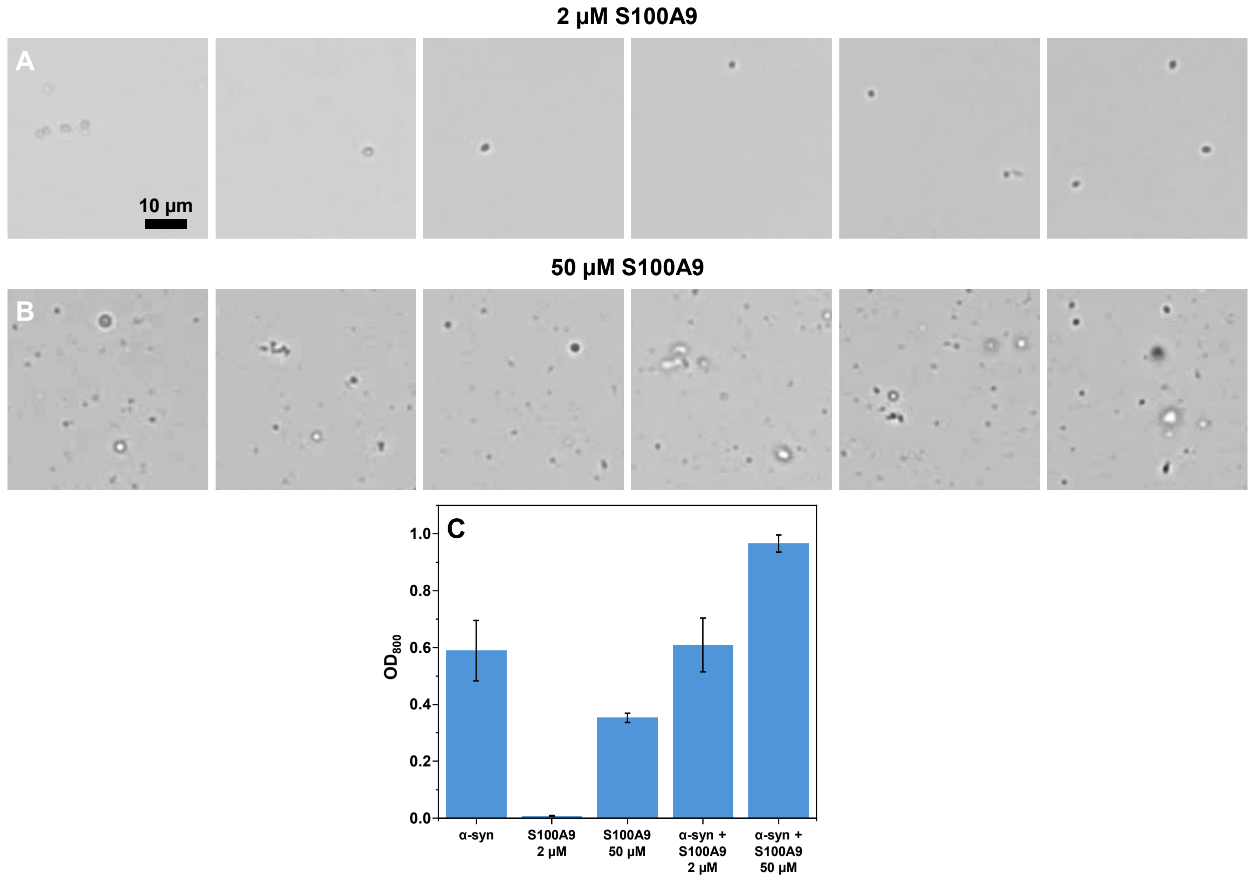

Figure S4. Brightfield microscopy images of unlabeled S100A9 droplet formation (A – 2 µM S100A9, B – 50 µM S100A9). Scale bar (10 µm) is identical for all images. Optical density (800 nm) of 200 µM α-syn, 2 µM S100A9, 50 µM S100A9 and protein mixtures under LLPS-inducing conditions (C).

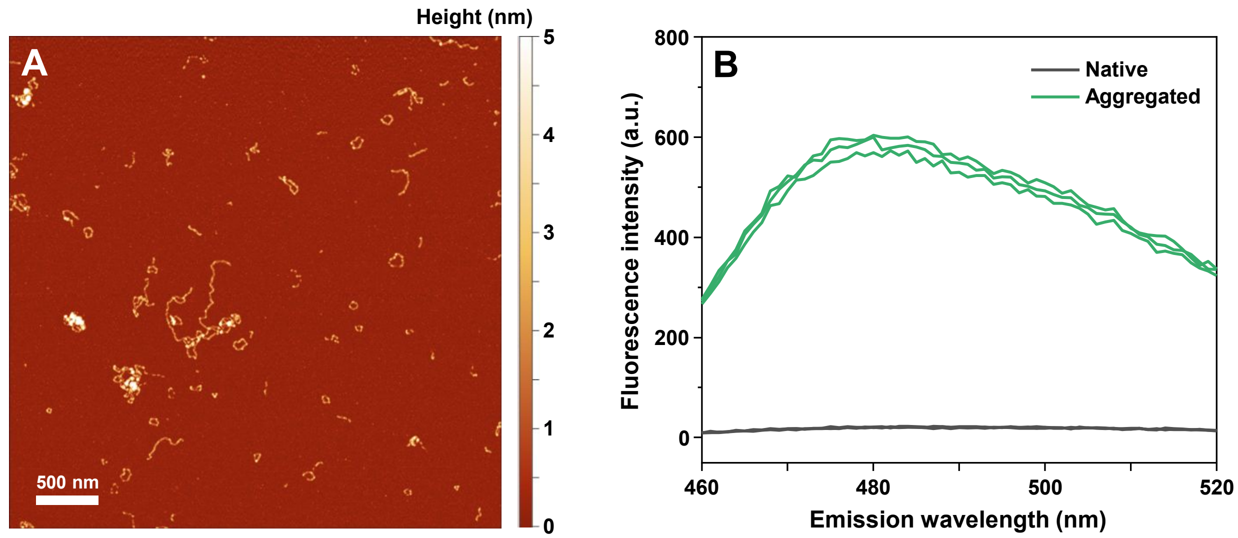

Figure S5. Atomic force microscopy image of S100A9 aggregates (A, scale bar is 500 nm) and fibril-bound ThT fluorescence emission spectra (B, n=3).

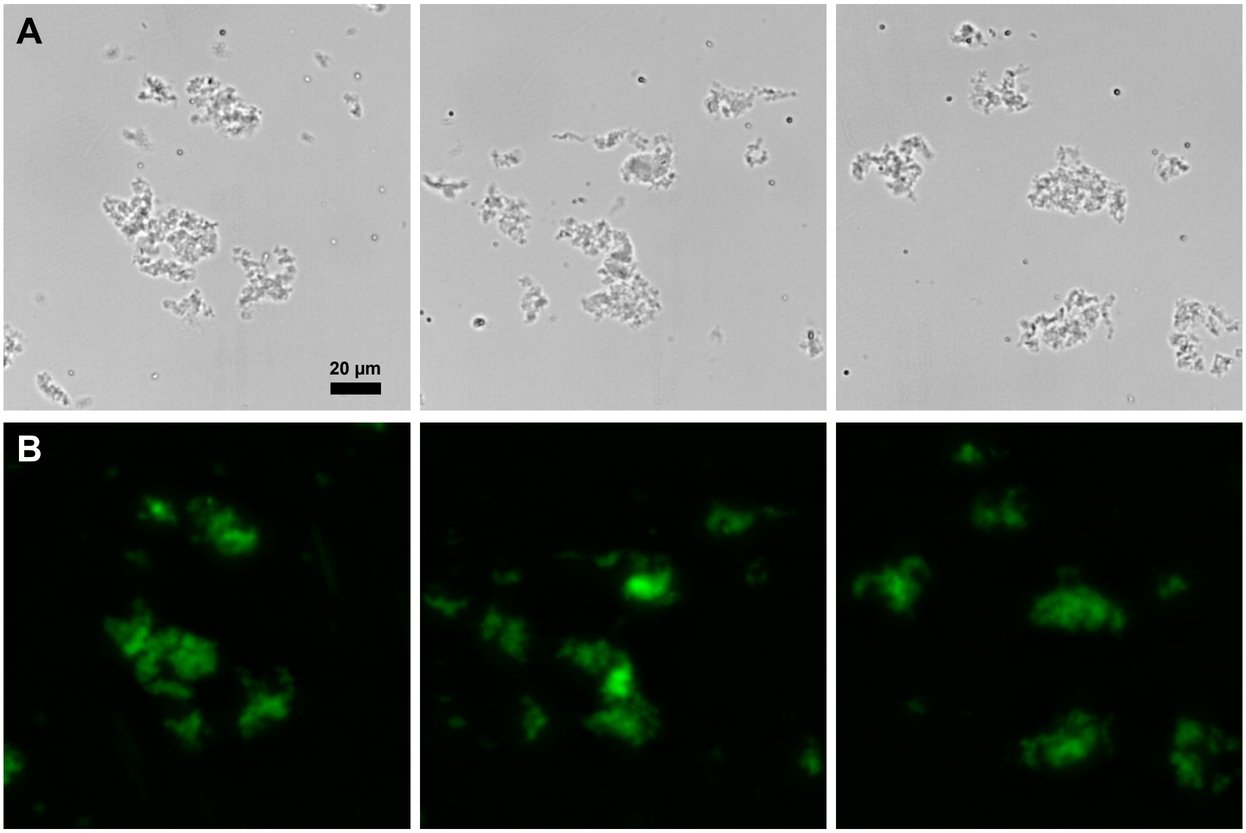

Figure S6. Brightfield and fluorescence microscopy images of S100A9 aggregates with 100 μM ThT. Brightfield microscopy images (Olympus IX83 microscope) of samples containing 50 μM S100A9 fibrils and 100 μM ThT (A, scale bar – 20 μm). Fluorescence microscopy images of the samples at the same exact positions (B, scale bar – 20 μm). All images were acquired after 10 minutes of sample incubation at 22°C. Imaging was conducted over a span of 15 minutes at the same temperature.

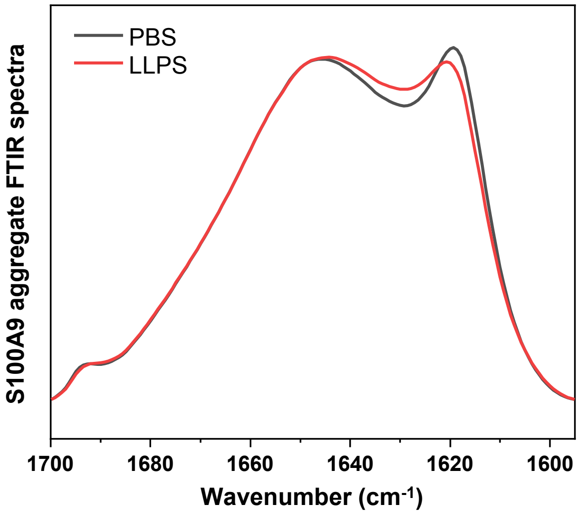

Figure S7. FTIR spectra of S100A9 fibrils prepared under non-LLPS (PBS, pH 7.4) and LLPS (20% PEG, PBS) conditions.

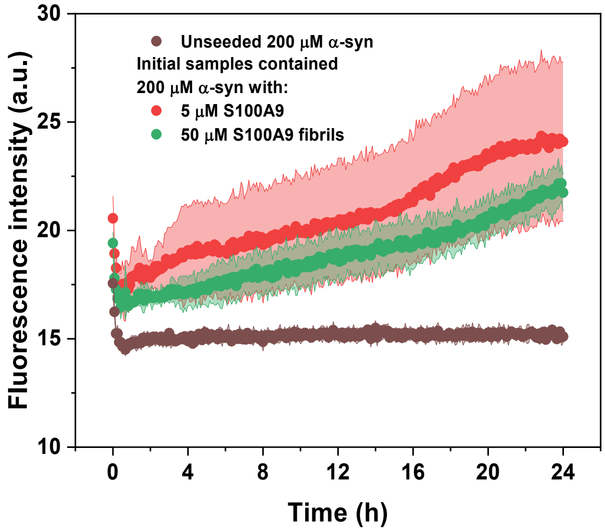

Figure S8. Second round of α-syn aggregate reseeding kinetics.

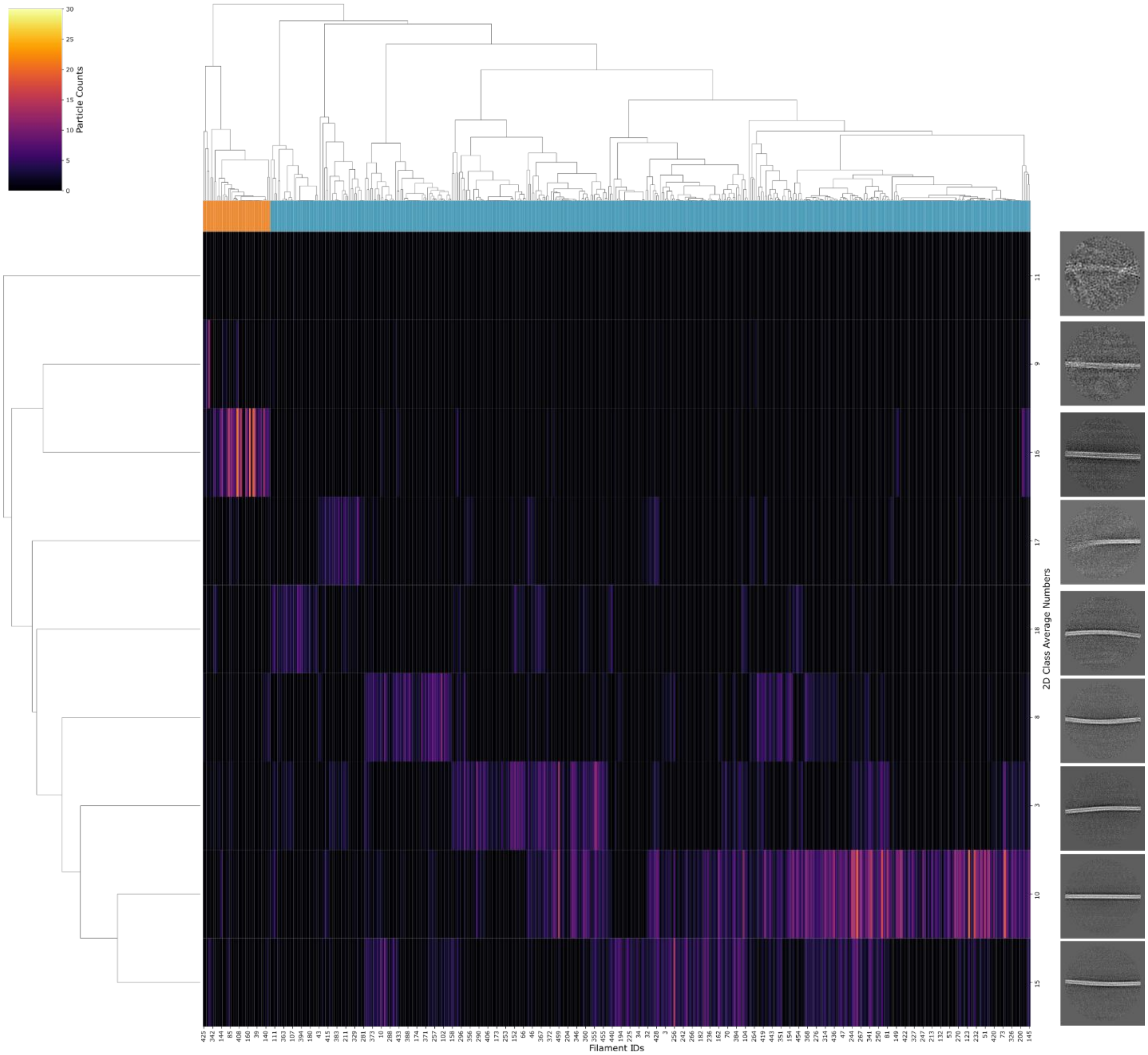

Figure S9. Hierarchical classification of α-syn (reseeded from α-syn LLPS) filament segments according to their assigned 2D class average number (vertical) and the picked filament ID (horizontal).

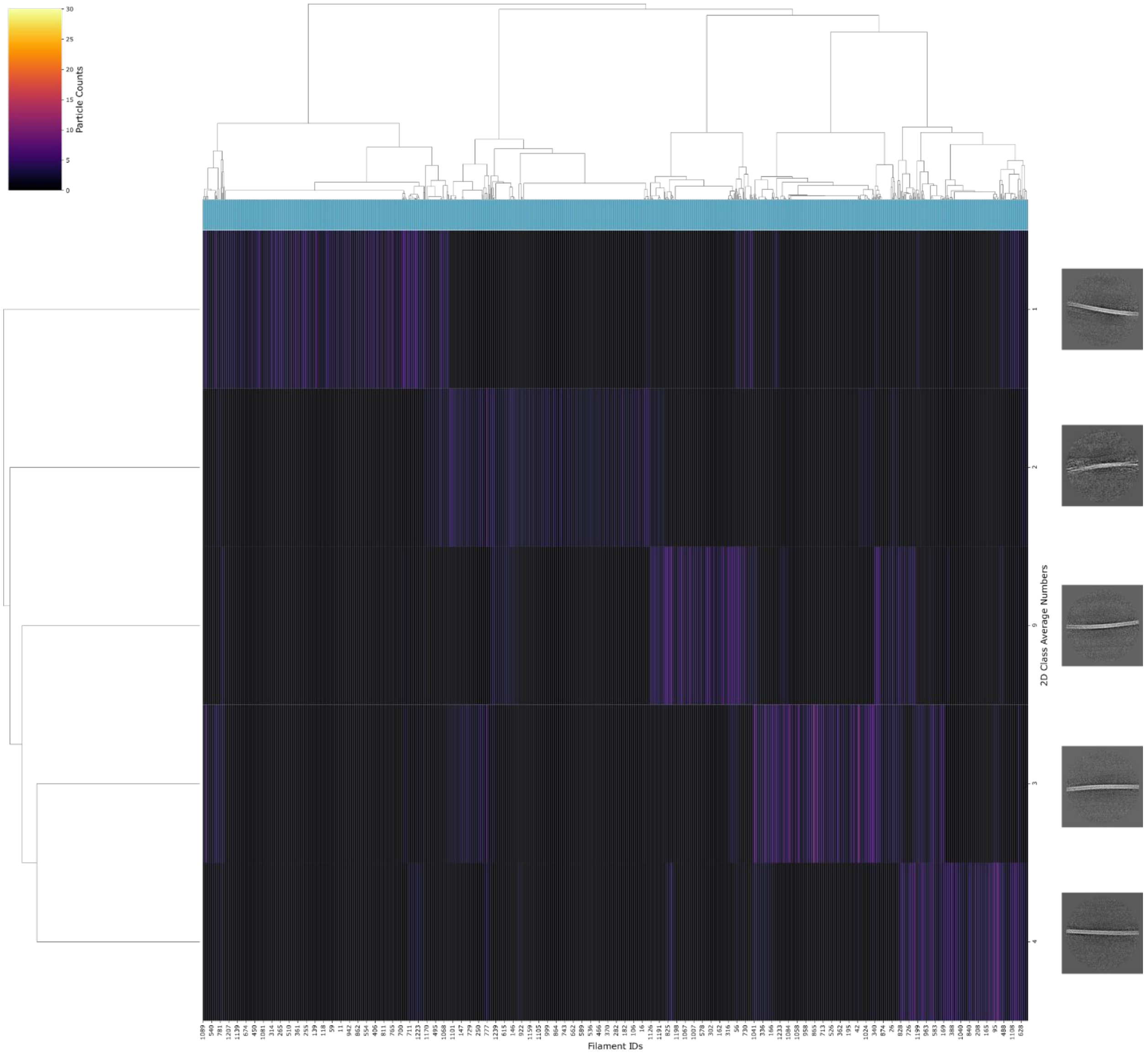

Figure S10. Hierarchical classification of α-syn (reseeded from α-syn + S100A9 LLPS) filament segments according to their assigned 2D class average number (vertical) and the picked filament ID (horizontal).

Primers	Sequence
DS105_S100A9_frw	GTGGTGGTGGTTCTGGTGGTGGTGGTTCTATGACTTGCAAAATGTCGCAGCTGG
DS105_S100A9_rev	GCGGATCCTTAGGGGGTGCCCTCCCCG
DS105_mCherry_frw	CGCATATGGTGAGCAAGGGCGAAGAAGATAAC
DS105_mCherry_rev	GAACCACCACCACCAGAACCACCACCACCCTTGTACAGCTCGTCCATGCCGCCG

Table S1. Primers used in this study

Name	α-syn (reseeded from α-syn LLPS)	α-syn (reseeded from α-syn +S100A9 LLPS)
**Data Collection**		
Pixel Size (Å)	1.1	1.1
Defocus range (nm)	-2.2 to –1.2	-2.2 to –1.2
Voltage (kV)	200	200
Camera	Falcon 3EC	Falcon 3EC
Microscope	Glacios	Glacios
Exposure time (s)	46.33	46.33
Number of frames	30	30
Total dose ($e^-/Å^2$)	30	30
**2D Classification**		
Micrographs	881	973
Picked fibrils	1158	8816
Box size (px)	1024	1024
Inter-box distance (Å)	69.09	69.09
Segments Extracted	4072	9204
Segments used for final 2D classification	4072	4060

Table S2. Statistics of Cryo-EM data collection and 2D classification.
